# Downexpression of HSD17B6 correlates with clinical prognosis and tumor immune infiltrates in hepatocellular carcinoma

**DOI:** 10.1186/s12935-020-01298-5

**Published:** 2020-06-03

**Authors:** Lei Lv, Yujia Zhao, Qinqin Wei, Ye Zhao, Qiyi Yi

**Affiliations:** 1 Teaching and Research Section of Nuclear Medicine, Anhui Medical University, 81 Meishan Road, Hefei, 230032 Anhui People’s Republic of China; 2 The First Affiliated Hospital of USTC, Division of Life Sciences and Medicine, University of Science and Technology of China, Hefei, 230031 Anhui People’s Republic of China

**Keywords:** HSD17B6, HNF4A, DNA methylation, Hepatocellular carcinoma (HCC), Androgen, Immune suppression

## Abstract

**Background:**

Hydroxysteroid 17-Beta Dehydrogenase 6 (HSD17B6), a key protein involved in synthetizing dihydrotestosterone, is abundant in the liver. Previous studies have suggested a role for dihydrotestosterone in modulating progress of various malignancies, and HSD17B6 dysfunction was associated with lung cancer and prostate cancer. However, little is known about the detailed role of HSD17B6 in hepatocellular carcinoma (HCC).

**Methods:**

Clinical implication and survival data related to HSD17B6 expression in patients with HCC were obtained through TCGA, ICGC, ONCOMINE, GEO and HPA databases. Survival analysis plots were drawn with Kaplan–Meier Plotter. The ChIP-seq data were obtained from Cistrome DB. Protein–Protein Interaction and gene functional enrichment analyses were performed in STRING database. The correlations between HSD17B6 and tumor immune infiltrates was investigated via TIMER and xCell. The proliferation, migration and invasion of liver cancer cells transfected with HSD17B6 were evaluated by the CCK8 assay, wound healing test and transwell assay respectively. Expression of HSD17B6, TGFB1 and PD-L1 were assessed by quantitative RT-PCR.

**Results:**

HSD17B6 expression was lower in HCC compared to normal liver and correlated with tumor stage and grade. Lower expression of HSD17B6 was associated with worse OS, PFS, RFS and DSS in HCC patients. HNF4A bound to enhancer and promoter regions of HSD17B6 gene, activating its transcription, and DNA methylation of HSD17B6 promoter negatively controlled the expression. HSD17B6 and its interaction partners were involved in androgen metabolism and biosynthesis in liver. HSD17B6 inhibited tumor cell proliferation, migration and invasion in liver cancer cells and low expression of HSD17B6 correlated with high immune cells infiltration, relative reduction of immune responses and multiple immune checkpoint genes expression in HCC, probably by regulating the expression of TGFB1.

**Conclusions:**

This study indicate that HSD17B6 could be a new biomarker for the prognosis of HCC and an important negative regulator of immune responses in HCC.

## Background

Liver cancer is the sixth most common cancer and the fourth leading cause of cancer-related deaths worldwide, with 700,000 annual deaths in recent years [1, 2]. It is also the second most lethal tumor with only 18% of 5-year survival [3]. Hepatocellular carcinoma (HCC) accounts for 75–85% of primary liver cancers [2, 4], and various pathogenic factors can lead to its occurrence and development, such as chronic hepatitis B or C virus infections and alcohol abuse. Patients with HCC have sustained hepatic inflammation, fibrosis, which cause a series of genetic and epigenetic events. Despite tremendous efforts in the past decades, the incidence of HCC is increasing rapidly, and patients with advanced stage HCC have very poor outcomes [1]. Advances in HCC treatment will depend on a better understanding of the precise molecular mechanism involved in HCC. Although HCC is a clinically and biologically heterogeneous malignancy, most HCCs share common features regarding genetic and epigenetic alterations. Identification of these common molecular alterations in HCC might provide a rational strategy for the development of effective molecular targeted therapy in HCC.

HSD17B6 is a gene located on chromosome 12q13.3 and encodes a protein with both oxidoreductase and epimerase activities, involved in steroid metabolism. The oxidoreductase activity can convert 3 alpha-adiol to dihydrotestosterone (DHT), while the epimerase activity can convert androsterone to epi-androsterone [5, 6]. DHT was closely related to the development of many tumors, including prostate cancer, breast cancer, endometrial cancer, lung cancer and colorectal cancer [7]. Polymorphisms in the HSD17B6 gene are associated with polycystic ovary syndrome (PCOS) and key clinical phenotypes of the disorder [8, 9]. Expression of HSD17B6 in prostate cancer samples with bone metastasis were significantly lower than that in non-metastatic counterparts [10], indicating the dysfunction of HSD17B6 in cancer metastases. It has also been identified to be associated with non-small-cell lung cancer [11] and its SNP was significantly associated with liver fibrosis risk [12]. However, little is known about the role of HSD17B6 in the pathogenesis of HCC.

In the present study, we evaluated the expression patterns, potential functions, and prognostic values of HSD17B6 in HCC in detail, and found that HSD17B6 was frequently down-regulated and predicted poor prognosis in HCC. Moreover, our study indicated that transcription of HSD17B6 was positively regulated by hepatocyte nuclear factor 4 alpha (HNF4A), a liver-enriched transcription factor. Besides, DNA hypermethylation of the HSD17B6 promoter also contributed a lot to the low expression of HSD17B6 in HCC. Moreover, downregulation of HSD17B6 affected the cell proliferation, migration, invasion, androgen metabolism and biosynthesis in HCC, and was associated with infiltration of tumor immune cells and expression of multiple immune checkpoint genes, indicating an important role in HCC development, progression and immune therapy.

## Methods

### Oncomine analysis

Oncomine gene expression array datasets (https://www.oncomine.org, an online cancer microarray database) were used to analyze the transcription levels of gene expression in normal liver and liver disease tissues. The mRNA expressions of HSD17B6 in clinical cancer specimens were compared with those in normal controls, using a Student’s t test to generate a p value.

### The Kaplan–Meier plotter

The prognostic value of HSD17B6 mRNA expression in TCGA-LIHC was evaluated using an online database, Kaplan–Meier Plotter (http://www.kmplot.com) [13], which contained gene expression data and survival information of liver cancer patients (http://kmplot.com/analysis/index.php?p=service&cancer=liver_rnaseq). To analyze the OS (Overall survival), PFS (Progression-free survival), RFS (Relapse-free survival) and DSS (Disease specific survival) of patients with HCC, patient samples were split into two groups by auto select best cutoff (high versus low expression) and assessed by a Kaplan–Meier survival plot, with the hazard ratio (HR) with 95% confidence intervals (CIs) and log rank p value.

### The cancer genome atlas and GTEx data

The cancer genome atlas (TCGA) had both sequencing and pathological data on 30 different cancers [14]. The liver hepatocellular carcinoma (The cancer genome atlas, TCGA-LIHC) dataset, including data from 438 cases with pathology reports, was selected for further analyses of HSD17B6 using Xena Functional Genomics Explorer, which is a visual exploration resource for both public and private omics data, supported through the web-based Xena Browser and multiple turn-key Xena Hubs. The Genotype Tissue Expression (GTEx) Project is a data resource and tissue bank established by the National Institutes of Health Common Fund, and total of 53 human normal tissues from nearly 1000 individuals have been studied by genomic and RNA sequencing. The GTEx Project was supported by the Common Fund of the Office of the Director of the National Institutes of Health, and by NCI, NHGRI, NHLBI, NIDA, NIMH, and NINDS. The data used for the analyses described in this manuscript were obtained from GTEx Portal V8 Release on 10/10/2019.

### The basic expression of protein in liver and cancer tissues

We used the online tool “The human protein atlas” (HPA, https://www.proteinatlas.org) to analyze the basic protein expression level of HSD17B6 in human liver and liver cancer tissues [15–17]. The HPA is Swedish-based program initiated in 2003 with the aim to map all human proteins in cells, tissues, and organs using the integration of various omics technologies including antibody-based imaging. We worked with version 19 in this study, which was launched in September 2019 [15, 17]. With the purpose of analyzing the protein expression differences of HSD17B6 in normal liver and liver cancer tissues in live cancer patients, immunohistochemistry pictures were downloaded from the Tissue Atlas and Pathology Atlas in HPA.

### Protein–protein interaction (PPI) network construction and gene function and pathway enrichment analysis

We used the search tool for the retrieval of interacting genes (STRING) database to analyze functional interactions between proteins [18]. We retrieved interactions with confidence scores greater or equal to 0.9 and screened the resulting PPI network for analysis. And, we then performed a functional enrichment analysis of these genes using STRING database.

### Computational deconvolution of infiltrating immune cells

In order to evaluate the correlation of the infiltrating immune cell subsets in HCC samples with the expression of HSD17B6, we performed the deconvolution analysis through two web-based tools, TIMER (http://timer.cistrome.org/) and xCell (http://xcell.ucsf.edu/), to infer the presence in TCGA LIHC and ICGC LIRI-JP datasets. TIMER could estimate the abundances of six immune cell types, including B cells, CD4^+^ T cells, CD8^+^ T cells, neutrophils, macrophages, and dendritic cells via “Estimation” modules. xCell could estimate the abundance scores of 64 cell types including 34 kinds of immune cells. The correlations between HSD17B6 expression and abundance scores of immune cells evaluated by Spearman’s correlation.

### HCCDB analysis

The HCCDB database (http://lifeome.net/database/hccdb/home.html) archived 15 public HCC gene expression datasets containing totally 3917 samples (13 GEO microarray datasets and two RNA-Seq datasets, TCGA-LIHC and ICGC LIRI-JP), serving as a one-stop online resource for exploring HCC gene expression with user-friendly interfaces [19]. HCCDB was used to analyze the expression levels of HSD17B6 and over survival in different datasets.

### Gene set enrichment analysis (GSEA)

Correlation coefficients between each gene and HSD17B6 expression generated using Spearman correlation analysis were enrolled to create the pre-ranked gene list. Then, a pre-ranked GSEA analysis was performed on GO-term database (c5.bp.v7.0.symbols.gmt) of Molecular Signatures Database (MSigDB) with the pre-ranked list. Gene sets with FDR < 0.25 and specifically enriched at the beginning and end of the ranked list were considered to be enrichment significance [20].

### Cell culture and lentivirus transfection

The HCC cell line HepG2, which were obtained from ATCC (catalog number HB-8065), were cultured in DMEM medium plus 10% fetal bovine serum (cat. no. 10099‑141; Thermo Fisher Scientific, Inc.) at 37 °C with 5% CO_2_. Antibiotic or antifungal agents were not used, to avoid the potential effects of these agents on gene expression and cytotoxicity assays. The recombinant lentivirus expressing HSD17B6 and empty lentivector expressing GFP alone were purchased from HanBio (Shanghai, China). To establish stable transfecting cell lines, HepG2 cells were transduced with lentivirus in the presence of 5 μg/ml polybrene. Puromycin was added into medium at the concentration of 2 mg/ml for stable cell line selection. After antibiotic selection for 2 weeks, stable transfected HepG2 cell lines were established.

### Cell proliferation assay

Cells in the logarithmic growth phase were seeded in 96-well plates at a cell density of 2 × 10^3^/well (in triplicate) to allow adhesion. At 0, 24, 48, 72 and 96 h, cells were incubated with 10 µl CCK‑8 at 37 °C for an extra 2 h. The optical density was then measured with a microplate reader (Tecan Group Ltd.) at 450 nm. The cells were then cultured with fresh medium until the next round of measurements. The mean and standard deviation of the triplet measurements were calculated and plotted.

### Wound‑healing assay

Confluent cells were serum‑starved in DMEM medium for 10–12 h and scratched using the tip of a 10-µl pipette. After being washed twice with PBS to remove non-adherent cells, the plates were added 500 µl DMEM medium plus 10% FBS. The wound area was photographed at 0, 24 and 48 h under an Olympus IX73 inverted microscope. A cell-free region was drawn and measured by CellSens Standard software (Olympus).

### Transwell invasion assay

A BioCoat™ Matrigel invasion chamber (cat. no. 40480; BD Biosciences) was used according to the manufacturer’s protocol. Briefly, 4 × 10^4^ cells were trypsinized, washed, suspended in 200 µl DMEM medium, and seeded in the upper portion of the invasion chamber. The lower portion of the chamber contained 500 µl of DMEM medium plus 10% FBS, which served as a chemo-attractant. After 48 h, the non-invasive cells were removed from the upper surface of the membrane with a cotton swab. The invasive cells on the lower surface of the membrane were stained with 0.1% crystal violet for 30 min at room temperature, and counted in four separate areas with an inverted microscope.

### Reverse transcription‑quantitative PCR (RT‑qPCR)

Total RNA was isolated from the cells at the logarithmic growth phase using TRNzol‑A + reagent (cat. no. DP421; Tiangen Biotech Co., Ltd.), and converted to cDNA with TGFB1/PD-L1 primer and β-actin primers using the HiScript II 1st Strand cDNA Synthesis kit (cat. no. R211‑01; Vazyme). The relative expression level of TGFB1/PD-L1 was normalized to β-actin using the 2^−ΔΔCt^ method. The sequences of the primers were listed as follows (5′ → 3′): TGFB1 forward, ACCAACTATTGCTTCAGCTC, reverse, TTATGCTGGTTGTACAGG; PD-L1 forward, GGCATTTGCTGAACGCAT, reverse, CAATTAGTGCAGCCAGGT; β-actin forward, GCCCATCTACGAGGGGTATG, reverse, GAGGTAGTCAGTCAGGTCCCG.

### 5‑Aza‑2′‑deoxycytidine treatment

HepG2 cells were treated with 50 mM 5‑aza‑2′‑deoxycytidine (5‑aza‑dC; cat. no. A3656; Sigma‑Aldrich; Merck KGaA) for 72 h with a change of culture medium every 24 h as previously described [21].

### Western blot analysis

Cells were lysed in a solution of 60 mM Tris–HCl, pH 6.8, 2.00% sodium dodecyl sulfate, 20.00% glycerol, 0.25% bromophenol blue, 1.25% 2-mercaptoethanol and heated at 100 °C for 10 min. Protein concentrations were determined using a BCA protein assay kit. After being separated by 10 or 12% SDS-PAGE, the protein (40-50 µg) was transferred to a PVDF membrane (cat. no. IPVH00010; EMD Millipore). The membranes were blocked in 1× PBS buffer containing 5% BSA (cat. no. A1933; Sigma‑Aldrich; Merck KGaA) and 0.05% Tween‑20 (cat. no. A100777; Sangon Biotech Co., Ltd.) for 1 h at room temperature, and then incubated with the following primary antibodies: HSD17B6 (rabbit anti‑human ployclonal; 1:1000; cat. no. 11855-1-AP; ProteinTech Group, Inc.); GAPDH (mouse anti‑human monoclonal; 1:2000; cat. no. 60004‑1‑Ig; ProteinTech Group, Inc.) overnight at 4 °C. The blots were washed with PBST three times for 10 min each and incubated with secondary antibodies anti‑rabbit IgG (1:3000; cat. no. SA00001‑2); and anti‑mouse IgG (1:3000, cat. no. SA00001‑1; both from ProteinTech Group, Inc.) for 1 h at room temperature. The target bands were revealed by SuperSignal West Pico PLUS chemiluminescence substrate (cat. no. 34580; Thermo Fisher Scientific, Inc.).

### Statistical analysis

Data are presented as mean ± standard error of the mean (SEM), and the statistical significance of differences between groups was assessed by student’s t-test. The correlation of gene expression was evaluated by Spearman’s correlation and statistical significance. In all figures, values of p ≤ 0.05 were considered as statistically significant with *(p < 0.05), **(p < 0.01), ***(p < 0.001), ****(p < 0.0001). All the analyses were undertaken using PRISM version 8.02 (GraphPad software, version 8, San Diego, CA. USA).

## Results

### HSD17B6 is down-regulated in patients with hepatocellular carcinoma

**Fig. 1:**
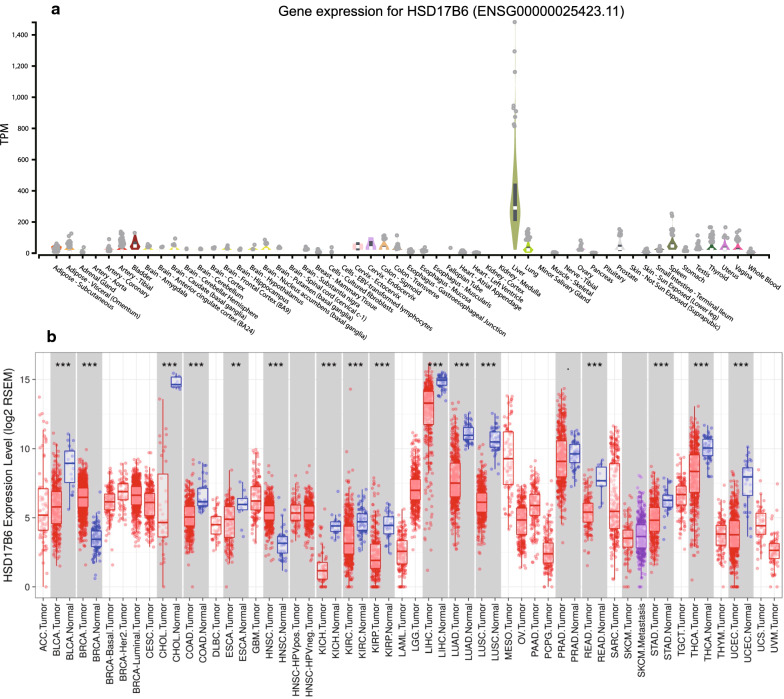
HSD17B6 mRNA expression levels in various types of human normal and cancer tissues. **a** Human HSD17B6 expression levels in different normal tissues from GTEx database; **b** Human HSD17B6 expression levels in different tumor and adjacent normal tissue from TCGA database. Distributions of gene expression levels are displayed using box plots, with statistical significance of differential expression evaluated using Wilcoxon test. P-value Significant Codes: *** < 0.001, ** < 0.01, * < 0.05

The male dominance in HCC occurrence suggested an important role of androgen and/or their receptors in the development of HCC [22]. A previous report suggested that androgen signals might suppress the cell invasion during the later stages of HCC [23]. HSD17B6 has both oxidoreductase and epimerase activities and is involved in androgen catabolism. It is mainly expressed in the liver (Fig. 1a) according to the GTEx, a data resource and tissue bank to study gene expression in multiple human normal tissues. To determine differences of HSD17B6 expression in tumor and normal tissues, the HSD17B6 mRNA levels in different tumors and normal tissues of multiple cancer types were analyzed in TCGA using the TIMER “DiffExp” module. HSD17B6 expression was significantly lower in most of tumor tissues, including LIHC (Liver hepatocellular carcinoma), compared to the normal tissues (p < 0.001, Fig. 1b). Higher expression of HSD17B6 was only observed in BRCA (Breast invasive carcinoma) and HNSC (Head and Neck squamous cell carcinoma) compared to the normal (Fig. 1b).

**Fig. 2:**
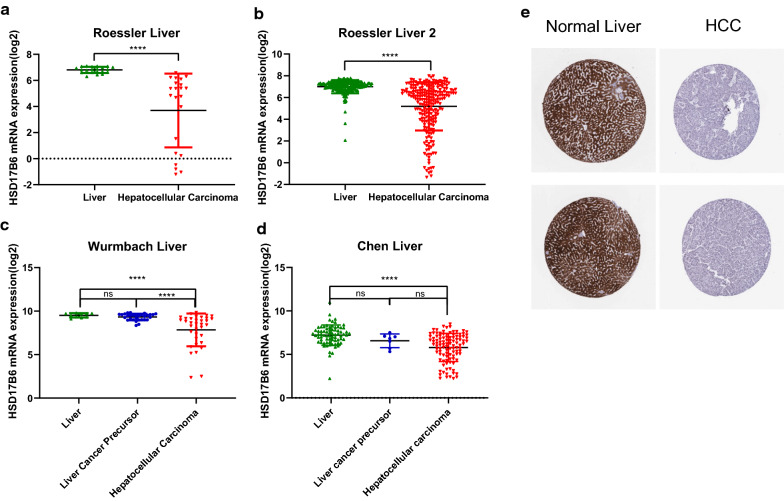
HSD17B6 expression levels in different HCC datasets. **a**–**d** Human HSD17B6 mRNA expression levels in HCC and adjacent normal tissue from GEO datasets. **e** The human protein atlas project shows representative immunohistochemical images from HSD17B6 in HCC compared with noncancerous liver tissues. The normal liver tissue of HSD17B6 was from two normal live samples (staining: high; intensity: strong;), and the HCC tissue was from a 66-year old female, HCC patient (patient ID: 82; staining: not detected; intensity: negative)

To further evaluate HSD17B6 expression in human liver cancers, the mRNA expression level of the HSD17B6 gene was examined using several other bioinformatics web resources. First, we analyzed four microarray datasets from the Oncomine Platform, and found that HSD17B6 transcript levels were significantly reduced in patients with HCC in all four datasets (p < 0.0001, Fig. 2a–d). Especially, in Wurmbach Liver and Chen Liver dataset, although HSD17B6 was downregulated in HCC versus normal liver tissue, its expression level in liver cancer precursors was not significantly reduced (Fig. 2c, d). HSD17B6 expression was also analyzed in multiple datasets with HCCDB tool, a one-stop online platform for exploring HCC gene expression [19]. Totally, 9 of 10 datasets showed that HSD17B6 expression in HCC tissues was significantly decreased compared with in adjacent/healthy tissues (Table 1). This trend was further verified by pathologic analyses. The IHC staining results from the Human Protein Atlas project suggested that HSD17B6 was strongly expressed in normal liver specimens (negative rate of 0%) but weakly expressed or not detected in HCC tissues (negative rate of 42%) (Fig. 2e). These results collectively suggest that reduced expression of HSD17B6 may promote hepatocarcinogenesis, acting as a tumor suppressor.

**Table 1:** Expression levels of HSD17B6 in different datasets

HCCDB ID	Source ID	P-value	Type	Sample numbers	Mean (log2)	STD (log2)
HCCDB1	GSE22058	7.25E−10	HCC	100	12.05	1.417
			Adjacent	97	13.02	0.232
HCCDB3	GSE25097	1.25E−27	HCC	268	9.786	5.504
			Adjacent	243	14.37	3.136
			Cirrhotic	40	9.1	5.075
			Healthy	6	14.72	1.735
HCCDB4	GSE36376	5.71E−23	HCC	240	10.51	2.007
			Adjacent	193	12.15	1.215
HCCDB6	GSE14520(GPL3721 Subset)	2.22E−27	HCC	225	9.463	2.176
			Adjacent	220	11.3	0.6076
HCCDB7	GSE10143	0.0007743	HCC	80	13.39	1.008
			Adjacent	82	13.83	0.4873
HCCDB11	GSE46444	0.01807	HCC	88	10.4	2.483
			Adjacent	48	11.33	1.949
HCCDB12	GSE54236	0.0005134	HCC	81	14.68	1.592
			Adjacent	80	15.37	0.6459
HCCDB16	GSE64041	0.003082	HCC	60	11.44	1.295
			Adjacent	60	12.05	0.866
HCCDB17	GSE76427	0.07784	HCC	115	12.45	2.032
			Adjacent	52	12.86	0.9313
HCCDB18	ICGC-LIRI-JP	4.57E−15	HCC	212	7.193	1.683
			Adjacent	177	8.275	0.8307

### HSD17B6 acts as a putative prognostic factor in hepatocellular carcinoma

**Fig. 3:**
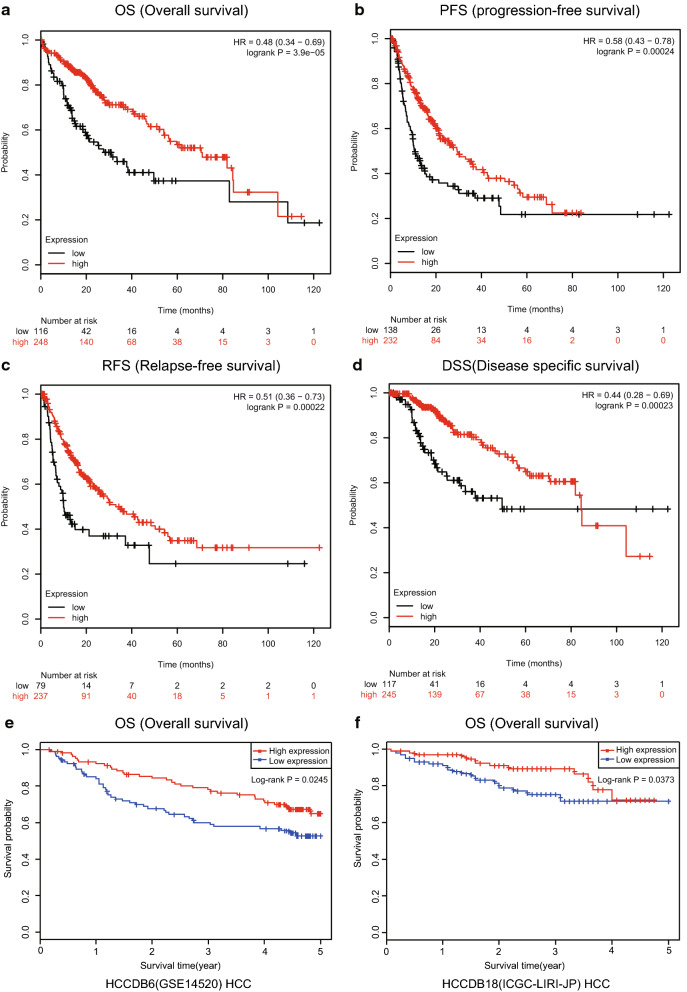
The association between HSD17B6 expression and survival rates of HCC patients. **a**–**d** Kaplan–Meier analysis of OS (Overall survival), PFS (Progression-free survival), RFS (Relapse-free survival) and DSS (Disease specific survival) in the TCGA-LIHC patients based on HSD17B6 expression. **e**, **f** Kaplan–Meier analysis of OS (Overall survival) in the patients with HCC based on HSD17B6 expression of GSE14520 and ICGC-LIRI-JP datasets using HCCDB

To the best of our knowledge, there have been no clear reports of the relationship between HSD17B6 expression and the clinical prognosis of HCC. As shown in plots using the Kaplan–Meier Plotter platform, an intriguing TCGA analysis tool, down-regulation of HSD17B6 expression was closely correlated with shorter OS (p = 3.9e−5), PFS (p = 0.00024), RFS (p = 0.00022) and DSS (p = 0.00023) (Fig. 3a–d), indicating a poorer prognosis with lower HSD17B6 level in HCC. Shorter OS was also observed in patients with lower expression of HSD27B6 in other two HCC datasets (p = 0.0245 for GSE14520 and p = 0.0373 for ICGC-LIRI-JP, respectively), analyzed by HCCDB tool (Fig. 3e, f).

**Fig. 4:**
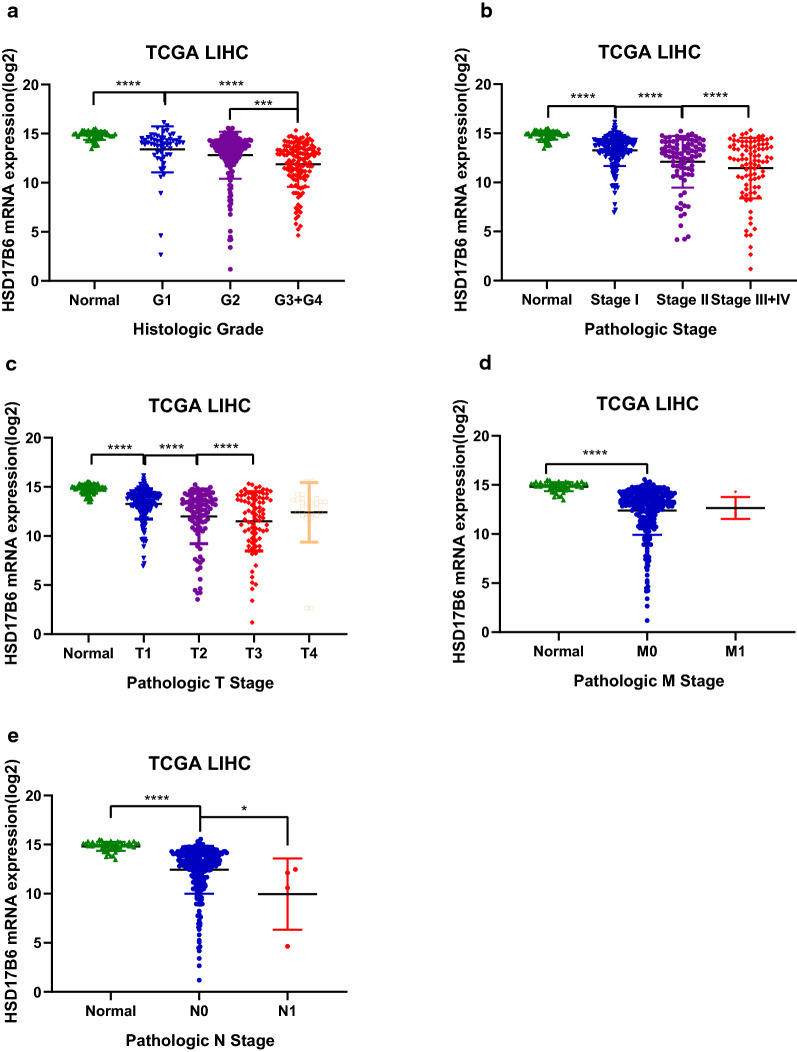
The association between HSD17B6 expression and clinical characteristics of HCC patients in TCGA LIHC datasets

**Fig. 5:**
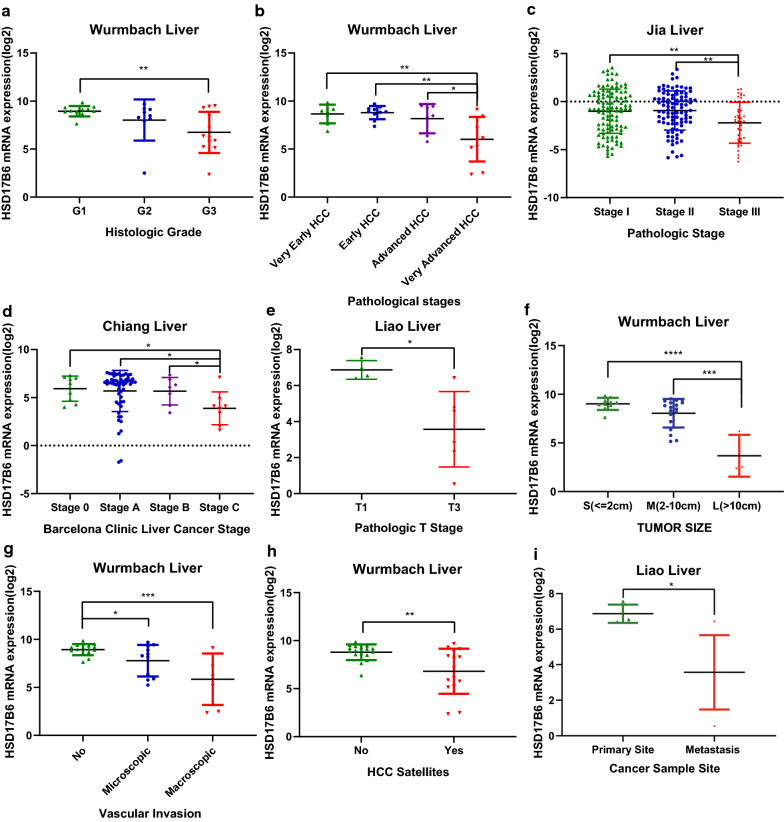
The association between HSD17B6 expression and clinical characteristics of HCC patients in multiple Oncomine datasets

To investigate what alterations in HCC relate to HSD17B6, we analyzed the association between HSD17B6 mRNA expression levels and the clinical characteristics of HCC patients in TCGA and four liver disease microarray datasets from the Oncomine Platform. Decreased HSD17B6 mRNA expression was associated with increased tumor grade (Fig. 4a, data from TCGA and Fig. 5a, data from Wurmbach liver dataset) and pathological stage (Fig. 4b, data from TCGA; Fig. 5b, c, data from Wurmbach Liver and Jia liver datasets). It was also associated with worse Barcelona Clinic Liver Cancer Stage, which is the most common staging system for liver cancer, in Chiang Liver dataset (Fig. 5d). Lower expression of HSD17B6 was associated with worse T stage (Fig. 4c, data from TCGA, and Fig. 5e, data from Liao Liver dataset), which refers to the size and extent of the main tumor. In accordance with this, lower expression of HSD17B6 was associated with larger tumor size in Wurmbach liver dataset (Fig. 5f). Lower expression of HSD17B6 was also associated with worse N stage in TCGA (Fig. 4e), which refers to the degree of spread to regional lymph nodes. Accordantly, HCC samples with more vascular invasion/satellites had significantly lower expression of HSD17B6 (Fig. 5g, h, data from Wurmbach liver dataset). Moreover, its expression level was lower in metastatic tumor tissues than in primary HCC (Fig. 5i).

Collectively, these results provide insight into the functional role of HSD17B6 in HCC and suggest that the reduced expression of HSD17B6 in HCC patients might be a valuable prognostic factor.

### Transcriptional regulation of HSD17B6 in hepatocellular carcinoma

**Fig. 6:**
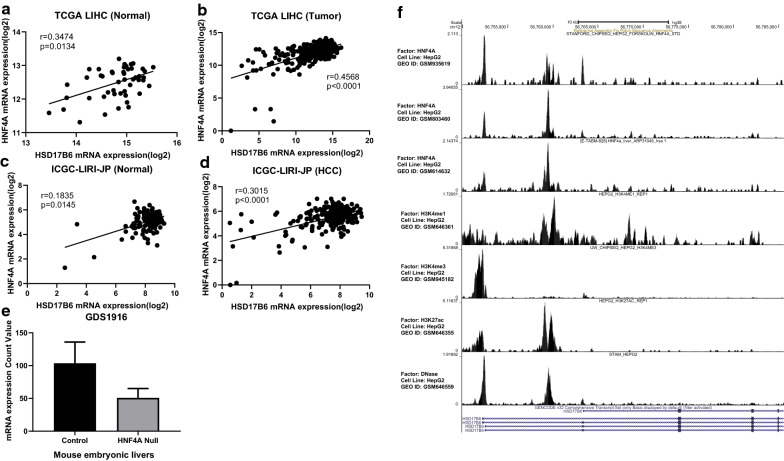
Expression of HSD17B6 is regulated by HNF4A. **a**–**d** Correlation of HSD17B6 expression with HNF4A expression in TCGA and ICGC-LIRI-JP liver datasets. **e** HNF4A knockout effect on HSD17B6 expression in the embryonic liver. **f** HNF4A binds to HSD17B6 enhancer and promoter region. Re-analysis of HNF4A ChIP-seq data available in CistromeDB platform. HNF4A chromatin immunoprecipitation was performed in HepG2 (liver cancer derived), and the presence of DNA peaks was evaluated in HSD17B6 enhancer and promoter regions

In order to shed light on mechanism involved in the regulation of HSD17B6, we first tried to find out transcription factors regulating HSD17B6. It has been recently reported that HNF4A, a liver-enriched transcription factor, could upregulate gene expression in hepatocytes by binding the enhancers of target genes [24]. There was a significant positive correlation between HSD17B6 and HNF4A transcript levels both in normal and HCC tissues from both TCGA and ICGC LIRI-JP datasets (Fig. 6a–d). Analysis of three liver disease microarray datasets from the Oncomine Platform confirmed this positive correlation (Additional file 1: Fig. S1). Additionally, analysis of HSD17B6 expression from GDS1916, a dataset form GEO database, showed that knockout of HNF4A in liver decreased the expression of HSD17B6 (Fig. 6e). Next, analyzing previously published, H3K4me1 [25], H3K4me3 [26], H3K27ac [25] and three HNF4A [27, 28] ChIP-seq datasets in HepG2 cells (a liver hepatocellular carcinoma cell line), we found the enrichment of modifications around HNF4A bound regions (HBRs) using CistromeDB [29, 30] (Fig. 6f). In particular, H3K4me1 and H3K4me3 mark enhancers and promoters respectively [31]. And H3K27ac is associated with the active state of both elements. Analysis of HNF4A ChIP-seq data revealed its preferential binding at first intron and promoter of HSD17B6 gene (Fig. 6f). Examination of HBRs demonstrated a high enrichment of H3K4me1 and H3K27ac flanking these regions (Fig. 6f), suggestive of active enhancers. The active status of these enhancers was further confirmed by examination of a HepG2 DNase CHIP data [32] (Fig. 6f), which showed very high signals, demonstrative of open chromatin at HBRs (Fig. 6f).

**Fig. 7:**
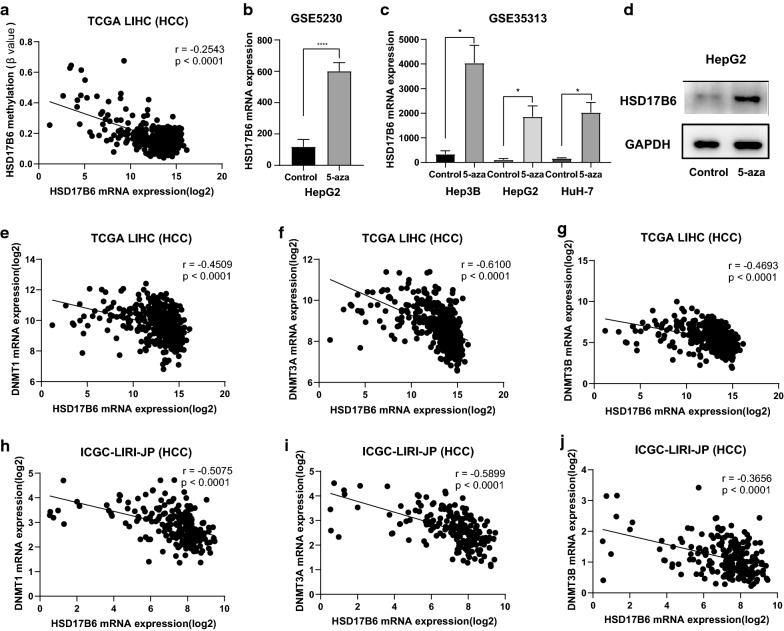
Expression of HSD17B6 is negatively regulated by DNA methylation. **a** Correlation of HSD17B6 expression with methylation status of HSD17B6 promoter in TCGA-LIHC datasets. **b**, **c** Effect of 5-aza treatment on the mRNA expression of HSD17B6 in HepG2 cells (**b** re-analysis of data form GSE5230), and three liver cancer lines (Hep3B, HepG2 and HuH-7) (**c** re-analysis of data form GSE35313). **d** Effect of 5-aza treatment on the protein expression of HSD17B6 in HepG2 cell. **e**–**j** Correlation of HSD17B6 expression with DNMTs (DNMT1, DNMT3A and DNMT3B) expression in TCGA-LIHC and ICGC-LIRI-JP liver datasets

**Fig. 8:**
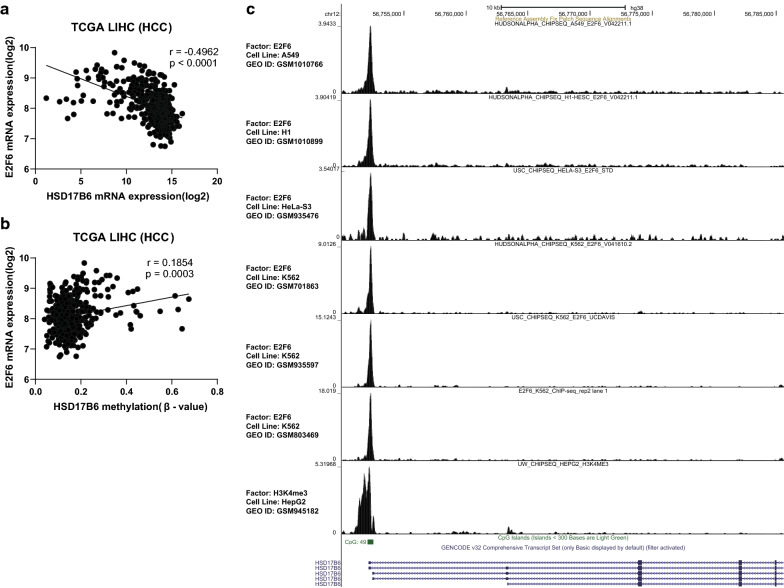
The methylation status of HSD17B6 promoter is dependent on E2F6. **a**, **b** Correlation of HSD17B6 expression with E2F6 expression in TCGA-LIHC dataset. **c** E2F6 binds to the promoter region of HSD17B6. Re-analysis of six different E2F6 ChIP-seq data available in CistromeDB platform. E2F6 chromatin immunoprecipitation was performed in four different human cancer-derived cell lines: A549 (lung carcinoma), H1 (embryonic stem cell), HeLa-S3 (cervical adenocarcinoma) and K562 (chronic myelogenous leukemia, including 3 ChIP-seq datasets from different research), and the presence of DNA peaks was evaluated in HSD17B6 promoter regions

DNA methylation is an epigenetic mechanism used by cells to control gene expression. We found that the methylation status of promoter of HSD17B6 was negatively correlated with expression of HSD17B6 in TCGA LIHC dataset (r = − 0.2543, Fig. 7a). Moreover, HSD17B6 expression was also negatively correlated with expression of DNA methyltransferases (DNMT1, DNMT3A and DNMT3B) in TCGA and ICGC LIRI-JP liver datasets (Fig. 7e–j). It has been reported that DNMT3B recruitment through E2F6 transcriptional repressor mediates DNA methylation and gene silencing [33]. ChIP-seq results from six independent investigations, accessed using CistromeDB, showed E2F6 binding at the HSD17B6 promoter (Fig. 8c), which harbors a CpG island (Fig. 8c). HSD17B6 expression was also negatively correlated with expression of E2F6 in TCGA and ICGC LIRI-JP liver datasets (Fig. 8a, b). Together, these results suggest that E2F6 might repress HSD17B6 expression by recruiting DNMT3B to HSD17B6 promoter. In addition, treatment with 5-aza-2′-deoxycytidine, a DNA-hypomethylating agent, upregulated the mRNA expression of HSD17B6 in live cancer cell lines (HepG2, Hep3B and HuH-7) based on the data from GSE5230 and GSE35313 datasets (p < 0.05, Fig. 7b, c). Confirming this, treatment with 5-aza-2′-deoxycytidine increased the protein level of HSD17B6 in HepG2 cells (Fig. 7d).

Taken together, the expression of HSD17B6 in HCC is regulated by both HNF4A and DNA methylation.

### HSD17B6 is Associated with the Androgen metabolic and biosynthetic processes

**Fig. 9:**
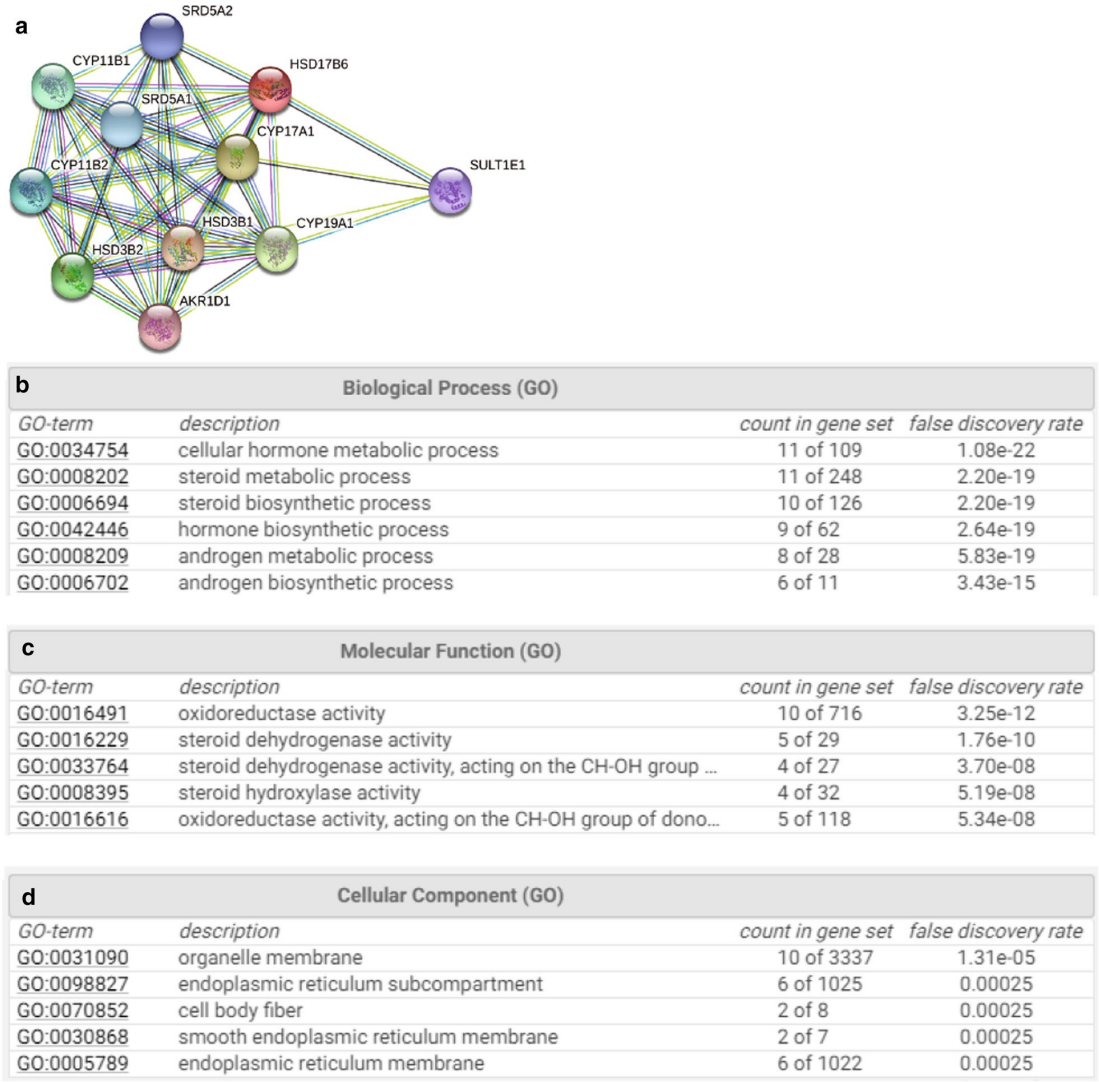
HSD17B6 Is associated with the androgen metabolic and biosynthetic. **a** Protein–protein interaction (PPI) network of HSD17B6. **b**–**d** Functional enrichment analysis of HSD17B6 and related proteins in three modules: **b** for GO (biological process), **c** for GO (molecular function) and **d** for GO (cellular component) analysis

Previous studies have demonstrated that androgen (a steroid hormone) and/or their receptors play important roles in the development of HCC [22, 23]. The protein encoded by HSD17B6 has both oxidoreductase and epimerase activities and is involved in androgen catabolism. We then used the Search Tool for the Retrieval of Interacting Genes (STRING) database to search for functional interaction proteins with HSD17B6, and found that 10 most related interaction proteins, which were RD5A2, CYP11B1, SRD5A1, CYP11B2, CYP17A1, HSD3B2, HSD3B1, CYP19A1, SULT1E1 and AKR1D1 (Fig. 9a). Further GO (Biological Process) analysis showed that all 11 proteins were involved in hormone (steroid) metabolic process. Among these 11 proteins, 9 were involved in androgen metabolic process, and 10 in steroid biosynthetic process. Particularly, 6 proteins (HSD17B6, SRD5A1, SRD5A2, HSD3B1, HSD3B2, and CYP17A1) were involved in androgen biosynthetic process (a process consisting of 11 proteins) (Fig. 9b). GO (Molecular Function) analysis showed that 10 of 11 proteins had oxidoreductase activity, 5 had steroid dehydrogenase activity, and 4 had hydroxylase activity (Fig. 9c). All these results indicate that HSD17B6 plays a very important role in androgen biosynthesis and androgen signaling pathway in HCC. Moreover, GO (Cellular Component) analysis shown that 6 of 11 proteins were located in endoplasmic reticulum (Fig. 9d). As endoplasmic reticulum stress contributes to the development and progression of HCC [34], HSD17B6 and its interaction proteins are likely linked to this process.

### HSD17B6 inhibits cell proliferation, migration, and invasion and correlates with immune response in HCC

To determine which biological processes (BP) are associated with HSD17B6, we searched for cellular signaling pathways altered in tumors with low HSD17B6 expression. We used Gene Set Enrichment Analysis (GSEA) to identify gene sets whose expression is enriched or depleted in low HSD17B6 expression tumors. This analysis was performed in TCGA LIHC and ICGC LIRI-JP datasets, and the common biological processes in these two datasets were used for further analysis.

**Fig. 10:**
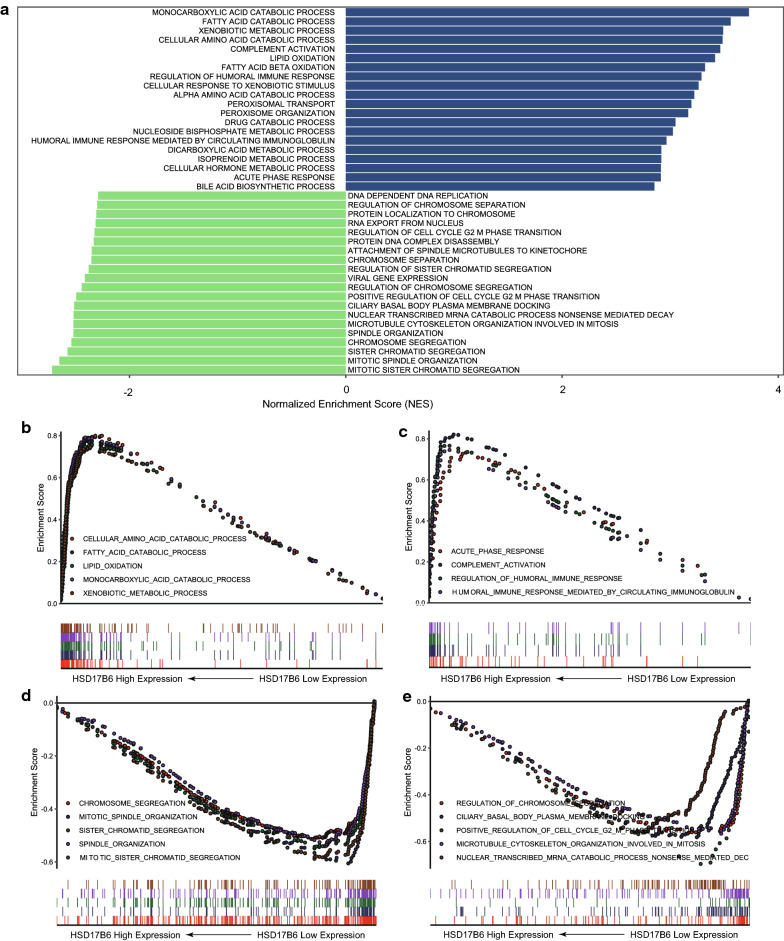
Gene set enrichment analysis (GSEA) analysis of HSD17B6. **a** The top twenty biological processes most positively/negatively correlating with expression of HSD17B6. **b** GSEA showed that HSD17B6 expression was positively correlated with biological processes implicated in metabolism. **c** GSEA showed that HSD17B6 expression was positively correlated with biological processes implicated in immune response. **d**, **e** GSEA showed that HSD17B6 expression was negatively correlated with biological processes implicated in mitosis and cell cycle regulation

**Fig. 11:**
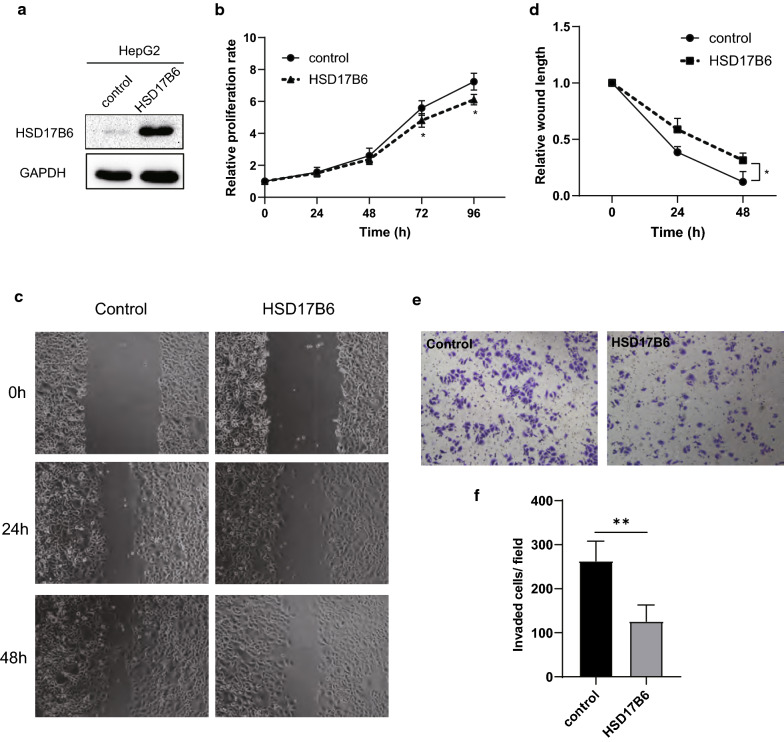
Effects of HSD17B6 overexpression on the cell proliferation, migration and invasion of HepG2 cells. **a** HSD17B6 expression in HepG2 was upregulated through lentivirus transfection. **b** CCK8 assay were performed to analyze the effect of HSD17B6 overexpression on HepG2 cell growth in vitro. Data are expressed as mean ± SD of 3 experiments (*p < 0.05). **c**, **d** Wound–healing assay was performed to analyze the effect of HSD17B6 overexpression on HepG2 cell migration (*p < 0.05). **e**, **f** Transwell assay was performed to analyze the effect of HSD17B6 overexpression on HepG2 cell invasion in vitro (**p < 0.01)

Eight of the ten biological processes most negatively correlating with expression of HSD17B6 were those implicated in mitosis and cell cycle regulation, such as chromosome segregation, mitotic spindle organization, and microtubule cytoskeleton organization involved in mitosis (Fig. 10a, d, e and Additional file 2: Table S1b). The levels of cyclin B1 (CCNB1) and CDC20, two markers of mitosis, were also negatively correlated with HSD17B6 expression in TCGA LIHC and ICGC LIRI-JP (Additional file 3: Fig. S2). To further explore the biological role of HSD17B6 in liver cancer, HSD17B6 expression was stably up-regulated in HepG2 cell through transfection (Fig. 11a). Cell proliferation was evaluated by CCK-8 assay. As shown in Fig. 11b, cell proliferation was significantly inhibited by HSD17B6 overexpression (p < 0.05) (Fig. 11b). These results suggest that overexpression of HSD17B6 could inhibit the proliferation of liver cancer cells. Moreover, the effects of HSD17B6 on the migrated and invasive abilities of liver cancer cells were assessed by wound‑healing assay and transwell assay respectively. Overexpression of HSD17B6 in HepG2 cells caused a significant reduction in cell migration (Fig. 11c, d) and cell invasion (Fig. 11e, f).

Two distinct groups of biological processes positively correlating with HSD17B6 expression emerged from this analysis. First, the biological processes associated with metabolism was significantly decreased in low HSD17B6 expression tumors (Fig. 10a, b and Additional file 2: Table S1a). Seven of the ten biological processes most positively correlating with expression of HSD17B6 were those implicated in metabolism, such as fatty acid catabolic process, xenobiotic metabolic process and lipid oxidation (Fig. 10a, b). Second, four of the twenty biological processes most positively correlating with expression of HSD17B6 were those implicated in immunity response, including complement activation, regulation of humoral immune response, humoral immune response mediated by circulating immunoglobulin and acute phase response (Fig. 10a, c). These indicated that in HCC with low expression of HSD17B6, the immune response to tumors was reduced.

### HSD17B6 is associated with tumor infiltrating immune cells and immune suppression in hepatocellular carcinoma

**Fig. 12:**
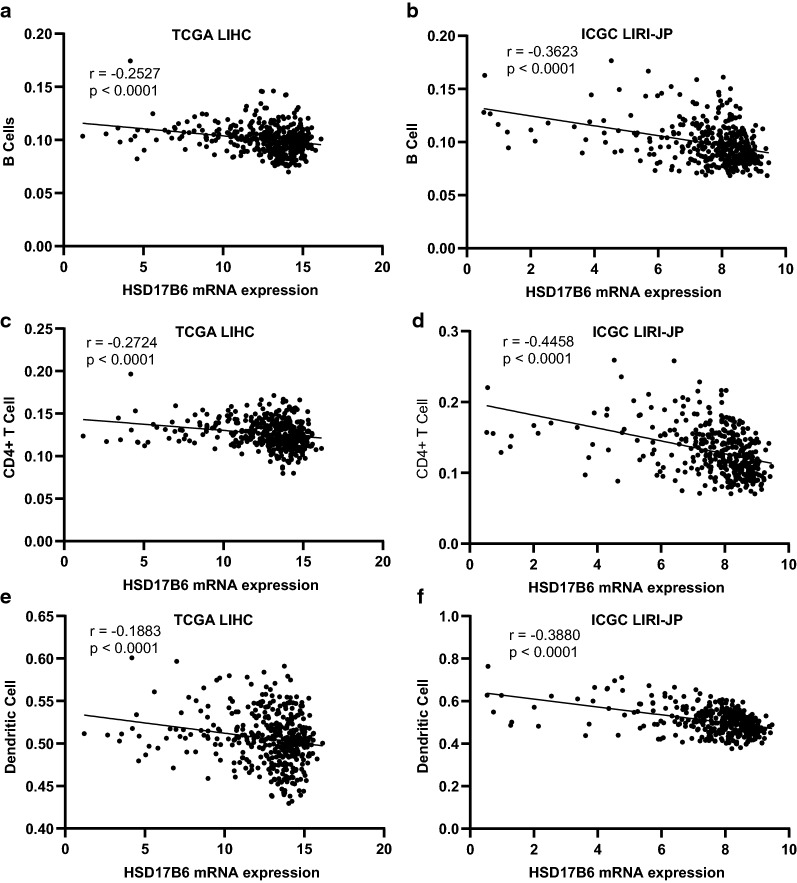
Correlation of HSD17B6 expression with infiltration level of immune cells by TIMER. **a**, **c**, **e** HSD17B6 expression negatively correlated with infiltrating levels of B cells, CD4^+^ T cells, and dendritic cells in TCGA LIHC dataset. **b**, **d**, **f** HSD17B6 expression negatively correlated with infiltrating levels of B cells, CD4^+^ T cells, and dendritic cells in ICGC LIRI-JP dataset

**Fig. 13:**
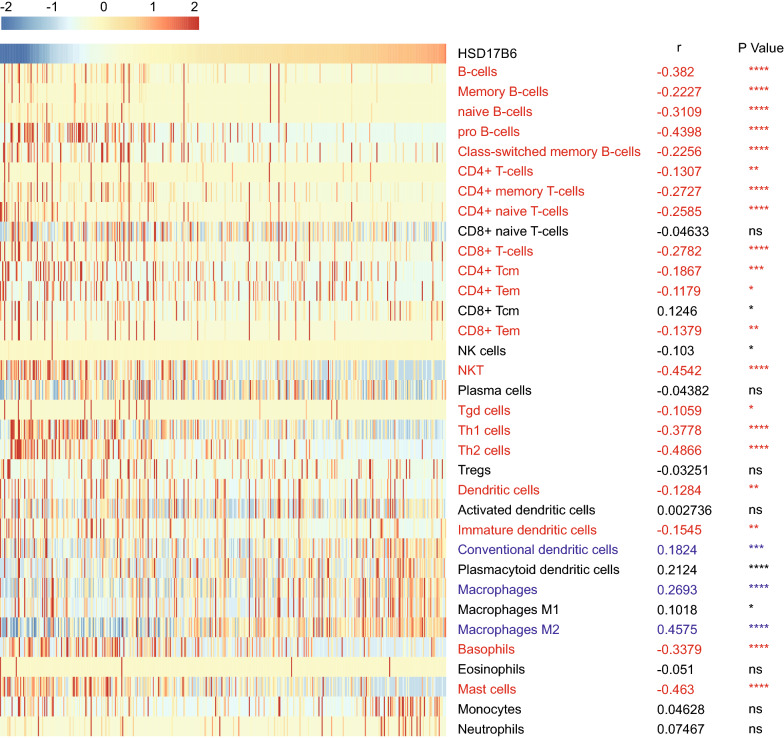
Correlation of HSD17B6 expression with infiltration level of immune cells by xCell in TCGA LIHC. Immune cells negatively correlating with HSD17B6 expression in both TCGA LIHC and ICGC LIRI-JP dataset were labeled in red, and immune cells positively correlating with HSD17B6 expression in both TCGA LIHC and ICGC LIRI-JP dataset were were labeled in blue. *(p < 0.05), **(p < 0.01), ***(p < 0.001), ****(p < 0.0001)

Tumor-infiltrating immune cells (TIICs) are a part of the complex microenvironment that regulates tumor development and progression [35]. Since recent studies revealed that TIICs were preferentially enriched and potentially clonally expanded in HCC [36], and accumulation of TIICs correlated with poor prognosis for HCC [37], we quantified the TIICs from RNA sequencing data through two widely used bioinformatics tools: “TIMER” and “xCell” [38, 39] in TCGA LIHC and ICGC LIRI-JP datasets. “TIMER” could estimate the abundances of six immune cell types, including B-cells, CD4^+^ T-cells, CD8^+^ T-cells, dendritic cells, macrophages and neutrophils. The analysis showed that infiltration level of B-cells, CD4^+^ T-cells and dendritic cells were negatively correlated with HSD17B6 expression in both TCGA LIHC and ICGC LIRI-JP datasets (Fig. 12). Further analysis using “xCell” also showed that infiltration of 20 kinds of immune cells, including B-cells, CD4^+^ T-cells and dendritic cells, was negatively correlated with HSD17B6 expression (Fig. 13, Additional file 4: Fig. S3). However, infiltration of 3 kinds of immune cells, including conventional dendritic cells, macrophages, macrophages M2, was positively correlated with HSD17B6 expression (Fig. 13, Additional file 4: Fig. S3).

**Fig. 14:**
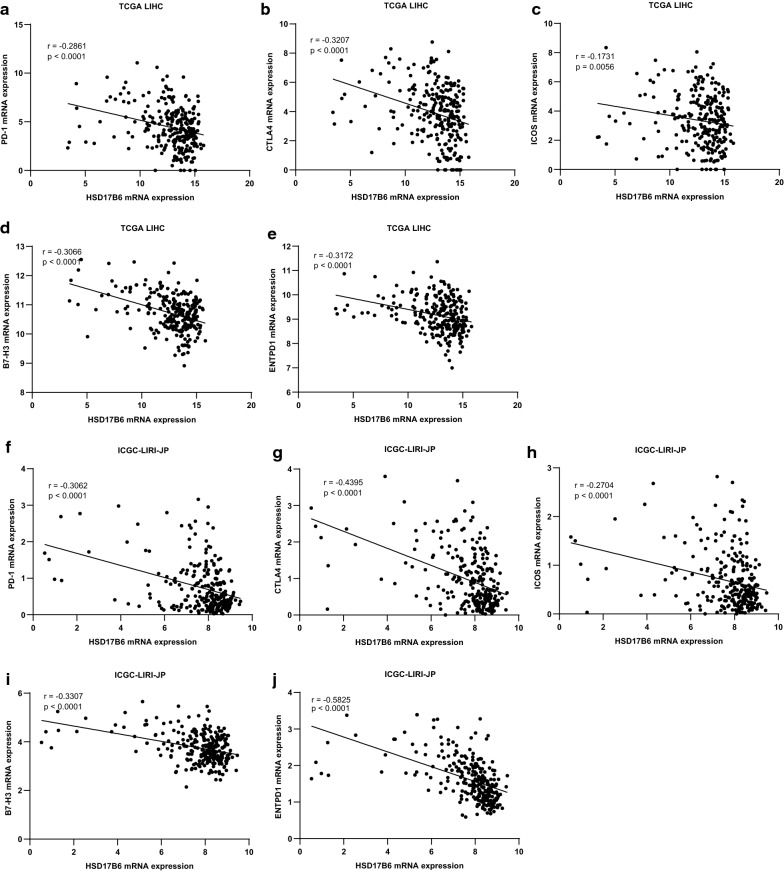
Correlation of HSD17B6 expression with immune checkpoint genes expression. **a**–**e** Expression of PD-1, CTLA4, ICOS, B7-H3 and ENTPD1 negatively correlated with HSD17B6 expression in TCGA LIHC. **f**–**j** Expression of PD-1, CTLA4, ICOS, B7-H3 and ENTPD1 negatively correlated with HSD17B6 expression in ICGC LIRI-JP

Tumors could evade immune responses by taking advantage of immune checkpoint genes, such as PD-1 and CTLA-4. We analyzed HSD17B6 and immune checkpoint genes expression in TCGA LIHC and ICGC LIRI-JP. HSD17B6 expression was negatively correlates with expression of PD-1(r = − 0.2861 in TCGA LIHC and r = − 0.3062 in ICGC), CTLA4(r = − 0.3207 in TCGA LIHC and r = − 0.4395 in ICGC), ICOS(r = − 0.1731 in TCGA LIHC and r = − 0.2704 in ICGC), B7-H3(r = − 0.3066 in TCGA LIHC and r = − 0.3307 in ICGC) and ENTPD1(r = − 0.3172 in TCGA LIHC and r = − 0.5825 in ICGC) (Fig. 14), suggesting that low HSD17B6 expression potentially plays an important role in mediating immune evasion.

**Fig. 15:**
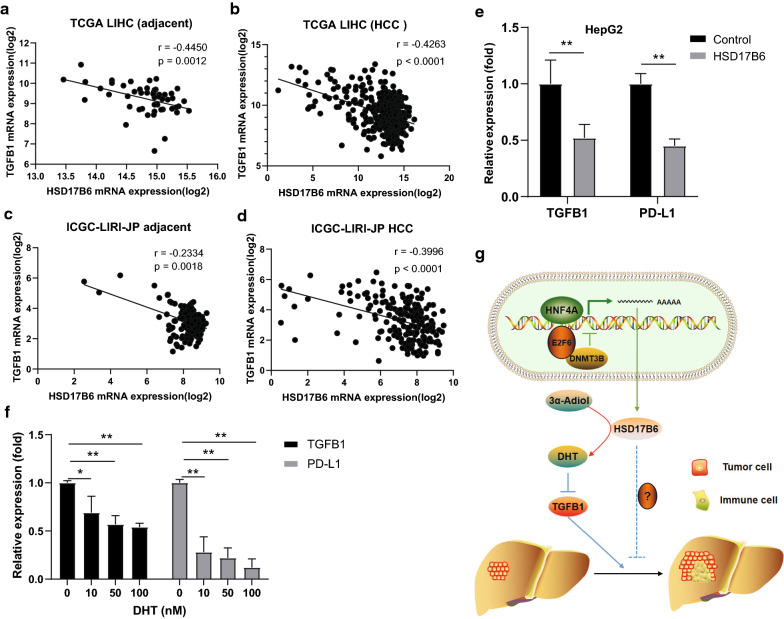
Downstream genes of HSD17B6. **a**–**d** Expression of TGFB1 negatively correlated with HSD17B6 expression in TCGA LIHC and ICGC LIRI-JP. **e** Expression of TGFB1 and PD-L1 was reduced in HepG2 with HSD17B6 overexpression. **f** Expression of TGFB1 and PD-L1 was reduced in HepG2 with DHT treatment. **g** Schematic diagram for this study

Transforming growth factor-β (TGF-β) has long been shown to play an essential role in establishing immune suppression and evasion [40, 41]. It has been reported that treatment with DHT, a product of HSD17B6, inhibited the expression of TGFB1 in prostate cancer [42]. Our analysis showed a significant negative correlation between HSD17B6 and TGFB1 transcript levels both in normal and HCC tissues from TCGA and ICGC LIRI-JP datasets (Fig. 15a–d). And both HSD17B6 overexpression and DHT treatment inhibited the expression of TGFB1 and PD-L1 in HepG2 cells (Fig. 15e, f).

## Discussion

This study was conducted to determine the feasibility of HSD17B6 as a potential biomarker in HCC patients. Our study is the first to analyze the differential expression of HSD17B6 in HCC tissues using different public datasets and examine its roles in cancer-related signaling pathways to identify its likely biological significance in HCC carcinogenesis. Using the TCGA, ICGC, Oncomine, GEO and HPA datasets, we have shown that HSD17B6 was significantly down-regulated in HCC tissues and that its expression level was negatively correlated with the OS, PFS, RFS and DSS of HCC patients. Using public CHIP datasets, we have proven that HSD17B6 was regulated by HNF4A and DNA methylation. Furthermore, we showed here that loss of HSD17B6 affected the androgen biosynthesis and androgen signaling pathway, promoted tumor cell proliferation, migration, invasion, immune cell infiltration and immune evasion partially through TGFB1.

HSD17B6 has both oxidoreductase and epimerase activities, involved in steroid metabolism. The oxidoreductase activity can convert 3 alpha-adiol to DHT, while the epimerase activity can convert androsterone to epi-androsterone [5, 6]. Previous studies have shown that polymorphisms of HSD17B6 gene are associated with polycystic ovary syndrome (PCOS) [8, 9]. For example, it was associated with reduced fasting glucose-insulin ratio and increased homeostasis model assessment and body mass index in the polycystic ovary syndrome group. The expression of HSD17B6 was significantly reduced in prostate cancer and it was undetectable in prostate cancers of Gleason grade higher than three [43]. In men receiving androgen-deprived therapy (ADT), patients showing biochemical progression had a higher HSD17B6 score than those without progression, suggesting that lower expression of HSD17B6 correlated with worse prognosis [44]. Its mRNA levels in castration resistant prostate cancer with bone metastatic samples were significantly lower than those without metastasis [10], indicating that HSD17B6 dysfunction involved in prostate cancer metastases. Downregulated HSD17B6 has also been identified to be associated with non-small-cell lung cancer [11], implying that it might be the key gene contributing to tumorigenesis or tumor progression in lung cancer [11]. Moreover, SNP of HSD17B6 was significantly associated with liver fibrosis risk [12]. In this study, using bioinformatics analyses of public datasets and experimentation, we have illustrated the key tumor suppressive roles of HSD17B6 in HCC samples (Fig. 15f).

DNA methylation status of CpGs, which is the best-characterized epigenetic mechanism, may play a pivotal role for downregulating HSD17B6 expression, as its expression was negatively correlated with the ratio of DNA methylation and expression of DNA methyltransferases (DNMT1, DNMT3A and DNMT3B) (Fig. 7e–j). And treatment with 5-aza, an inhibitor of DNA methylation, upregulated the HSD17B6 mRNA and protein expression of liver cancer cells (Fig. 7b–d). Actually, viral infection, chronic inflammation, and oxidative stress have been shown to affect DNMTs expression in HCC [45].Viral oncoproteins, such as the HCV core and the HBx protein of HBV, upregulate DNMT1 and DNMT3B [45]. HBx can directly interact with DNMT3A to induce abnormal hypermethylation at tumor suppressor gene (TSG) promoters [45]. Besides, HNF4A is also involved in regulation of HSD17B6, as their expression was positively correlated, and HNF4A binds the enhancer and promoter of HSD17B6. However, it is still unknown whether hypermethylation in the promoter region of HSD17B6 reduced the binding of HNF4A in HCC. Further studies are needed to more comprehensively explore the detailed molecular mechanisms of this altered biomarker in the progression of HCC.

In this study, biological pathway analyses had revealed clearly that the pathways related to androgen metabolic and biosynthetic processes in liver were the important pathways modulated by HSD17B6 (Fig. 9). Actually, cirrhotic patients with HCC had significantly lower plasma concentrations of several kinds of androgen including DHT, which was produced by HSD17B6, than patients with cirrhosis alone [46]. Moreover, high levels of DHT could induced cell cycle arrest and apoptosis in human liver cells by activation of PKR/eIF2alpha signaling cascade [47], and DHT gives growth advantage to cells by inhibiting TGF-beta-induced apoptosis in rat hepatocellular carcinoma cells [48]. However, another study showed that DHT could enhance HCC cell growth and apoptotic resistance [49]. Some previous reports confirmed the key roles of androgen signaling in the etiology of human cancers, including liver cancer [50]. Results from different studies indicated that androgen signaling might promote the initiation and development of HCC during the early stage [51], yet might suppress the cell invasion during the later stages of HCC [23]. These results and our study suggested that HSD17B6 contributed to HCC development by affecting androgen signaling pathway. Further research is needed to elucidate the detailed mechanisms.

Recent researches have revealed that tumor-infiltrating immune cells (TIICs) could regulates tumor development and progression [35]. Moreover, TIICs are preferentially enriched and potentially clonally expanded in HCC [36], and accumulation of TIICs correlates with poor prognosis for HCC [37]. Accordingly, we found that HSD17B6 expression in liver cancer was significantly negatively correlated with multiple immune cell infiltration, such as dendritic cells, CD4^+^ T cells, and B cells (Figs. 12, 13). Interestingly, recent studies shown that androgen receptor expression inversely correlates with immune cell infiltration in breast cancer [52], and low level of DHT could promote the infiltration of CD8^+^ T cells in prostate of benign prostatic hyperplasia (BPH) tissues [53]. What’s more, TGF-β promoted infiltration of immune cells and cancer-associated fibroblasts in the tumor microenvironments [54], and our present study showed that HSD17B6 could inhibit the expression of TGFB1 (Fig. 15). This may explain the negative correlation between the expression of HSD17B6 and the infiltration of immune cells.

The acquisition of genetic and epigenetic changes in cancer would lead to the expression of novel antigens, which are recognized by immune system, enabling the elimination of many nascent tumors [55]. Thus, immune evasion is one of the hallmarks of tumors including hepatocellular carcinoma [56, 57]. The most studied and clinically relevant immunosuppressive factors implicated in HCC are CTLA-4, PD-1, PD-L1 and the immunosuppressive cytokine TGF-β [55, 58–62]. Immune checkpoint proteins, including CTLA-4, PD-1, and PD-L1, could inhibit the activities of multiple immune cells, such as CD8^+^ T cells, natural killer cells and dendritic cells [63, 64]. TGF-β is a crucial cytokine to inhibit the expansion and function of many components of the immune system. It is central to tumor immune evasion and poor responses to cancer immunotherapy [64–66]. For example, TGF-β could upregulate expression of some immune checkpoint genes, such as TIM-3, CTLA-4, B7-H3, ENTPD1(CD39) and PD-1/PD-L1 [67–73]. And TGF-β1 is the most relevant member of the TGF-β family for immune regulation [41]. For example, TGF-β1 was shown to mediate tumor immune evasion from adaptive immunity [74]. Our present study has shown that HSD17B6 overexpression or treatment with its product DHT could inhibit the expression of TGFB1 and PD-L1 (Fig. 15). Furthermore, HS17B6 expression was positively correlated with immune response pathways in HCC (Fig. 10a, c). These data indicated that low expression of HSD17B6 promoted immune evasion through upregulating the expression of TGFB1 in HCC tumor microenvironment.

Our results must be carefully explained with great caution. There are some limitations in the present study. First, we showed here that low expression of HSD17B6 led to high expression of TGFB1 through DHT, and then promoted tumor development in HCC. However, the protein encoded by HSD17B6 has both oxidoreductase and epimerase activities. The oxidoreductase activity can convert 3 alpha-adiol to DHT, while the epimerase activity can convert androsterone to epi-androsterone. Further experiments should be carried out to determine which genes other than TGFB1 are affected by DHT and epiandrosterone, and their role in hepatocarcinogenesis. Second, we used bioinformatics tools to calculate the proportion of various immune cells in liver cancer samples. Further experiments, such as immunohistochemistry (IHC), immune fluorescence (IF), and flow cytometry, are needed to confirm these calculations.

In the present study, we identified HSD17B6 as a promising molecular marker with prognostic value in HCC. Analysis of the public liver cancer data provides a meaningful method to screen key genes associated with the onset of human malignant diseases in the future. Moreover, HSD17B6, could be a novel biomarker for the biological behavior of HCC, serving as a putative tumor suppressor gene.

## Conclusions

In conclusion, the data presented in this study show that HSD17B6 is frequently down-regulated in HCC. Moreover, our study indicated that transcription of HSD17B6 was positively regulated by hepatocyte nuclear factor 4 alpha (HNF4A), a liver-enriched transcription factor. Besides, DNA hypermethylation of the HSD17B6 promoter also contributed a lot to the low expression of HSD17B6 in HCC. HCC with low expression of HSD17B6 has worse tumor stage and prognosis. HSD17B6 may inhibit the expression of TGFB1 or other genes, and then inhibit tumor cell proliferation and invasion, immune cell infiltration and immune evasion (Fig. 15g). Taken together, this study demonstrates that HSD17B6 is an important player of liver carcinogenesis and helps better understanding of the molecular pathways involved in HCC development. The findings presented in this study add novel insight into the regulation of immune infiltration and suppression in HCC. We hope that it can help to make a better diagnosis and also help to predict the prognosis which might provide essential information regarding personalized treatment decisions for individual patients, especially for immune therapy.

## Supplementary information

**Additional file 1: Fig. S1.** Correlation of HSD17B6 expression with HNF4A expression in three Oncomine liver datasets.

**Additional file 2: Table S1.** List of top 20 common biological processes correlating with expression of HSD17B6 in TCGA LIHC and ICGC LIRI-JP datasets.

**Additional file 3: Fig. S2.** Correlation of HSD17B6 expression with CCNB1/CDC20 expression in TCGA LIHC and ICGC-LIRI-JP datasets.

**Additional file 4: Fig. S3.** Correlation of HSD17B6 expression with infiltration level of immune cells by xCell in ICGC LIRI-JP dataset.

## Data Availability

The datasets used and/or analyzed during the current study are available from the corresponding author on reasonable request.

## Abbreviations

HSD17B6: Hydroxysteroid 17-beta dehydrogenase 6
HCC: Hepatocellular carcinoma
TCGA: The cancer genome atlas
ICGC: International Cancer Genome Consortium
GEO: Gene expression omnibus
HPA: The human protein atlas
DHT: Dihydrotestosterone
PCOS: Polycystic ovary syndrome
HNF4A: Hepatocyte nuclear factor 4 alpha
GTEx: The genotype tissue expression
PPI: Protein–protein interaction
STRING: Search tool for the retrieval of interacting genes
GO: Gene ontology
TIICs: Tumor-infiltrating immune cells
DHT: Dihydrotestosterone
