# Correction to: Downexpression of HSD17B6 correlates with clinical prognosis and tumor immune infiltrates in hepatocellular carcinoma

**DOI:** 10.1186/s12935-020-01575-3

**Published:** 2020-10-02

**Authors:** Lei Lv, Yujia Zhao, Qinqin Wei, Ye Zhao, Qiyi Yi

**Affiliations:** 1 Anhui Provincial Cancer Hospital, West Branch of the First Affiliated Hospital of USTC, Division of Life Sciences and Medicine, University of Science and Technology of China, Hefei, 230031 Anhui People’s Republic of China; 2 Teaching and Research Section of Nuclear Medicine, Anhui Medical University, 81 Meishan Road, Hefei, 230032 Anhui People’s Republic of China

## Correction to Cancer Cell Int (2020) 20:210 http://doi.org/10.1186/s12935-020-01298-5

Following publication of the original article [1], we were notified of a few changes in the author affiliations. The correct affiliations for the authors are listed below:

Lei Lv^1,†^, Yujia Zhao^2,†^, Qinqin Wei^2^, Ye Zhao^2^, Qiyi Yi^2,*^

^1^Anhui Provincial Cancer Hospital, West Branch of the First Affiliated Hospital of USTC, Division of Life Sciences and Medicine, University of Science and Technology of China, Hefei 230031, Anhui, People’s Republic of China.

^2^Teaching and Research Section of Nuclear Medicine, Anhui Medical University, 81 Meishan Road, Hefei 230032, Anhui, People’s Republic of China.
