# Supplementary figures and images for: Downexpression of HSD17B6 correlates with clinical prognosis and tumor immune infiltrates in hepatocellular carcinoma
Source: Cancer Cell Int. 2020 Jun 3;20:210. doi: 10.1186/s12935-020-01298-5 (PMC7268300; doi:10.1186/s12935-020-01298-5)

**a****Chiang Liver**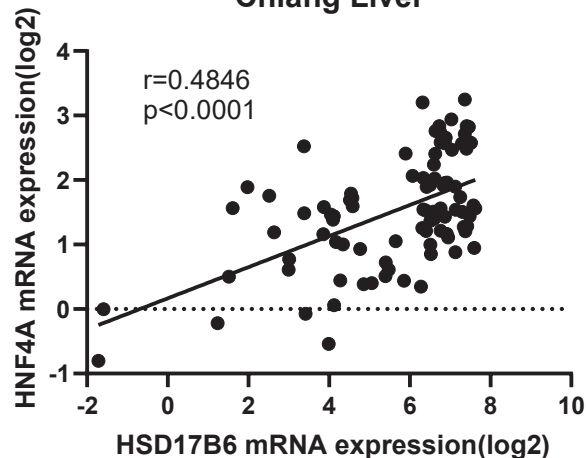**b****Woo Liver**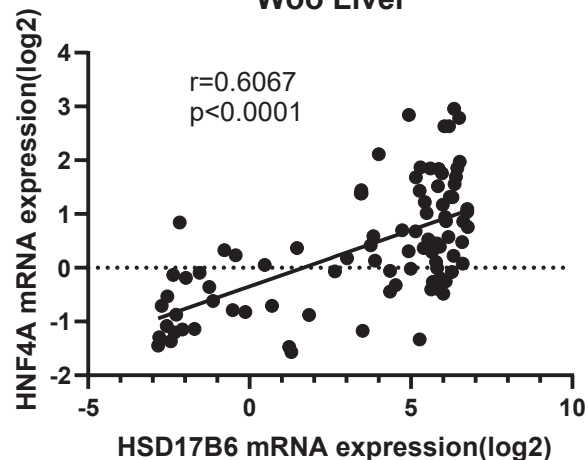**c****Roessler Liver 2(Normal)**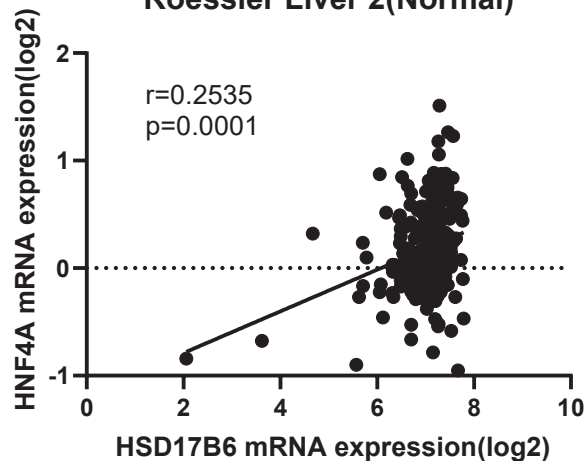**d****Roessler Liver 2(HCC)**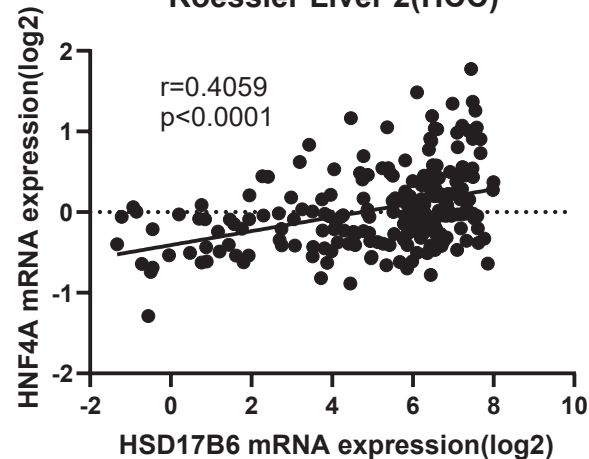

Supplement: Supplementary file 1 — Additional file 1: Fig. S1. Correlation of HSD17B6 expression with HNF4A expression in three Oncomine liver datasets. [file 12935_2020_1298_MOESM1_ESM.pdf]

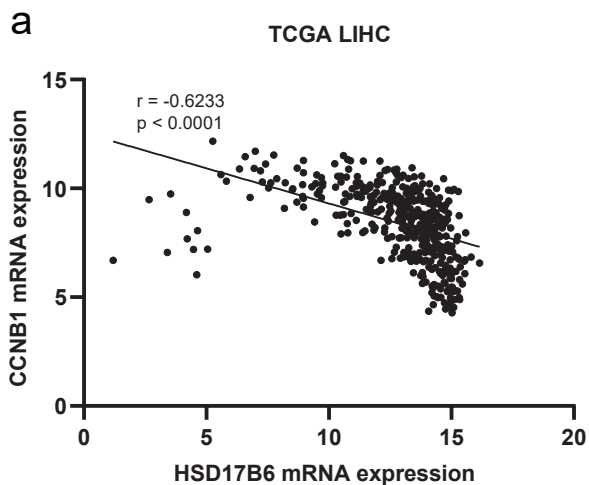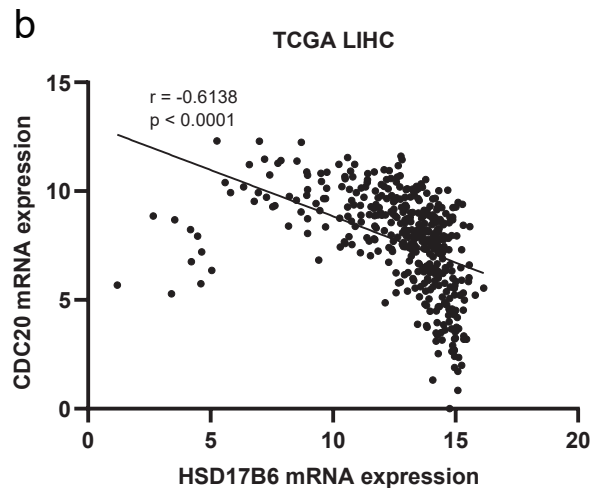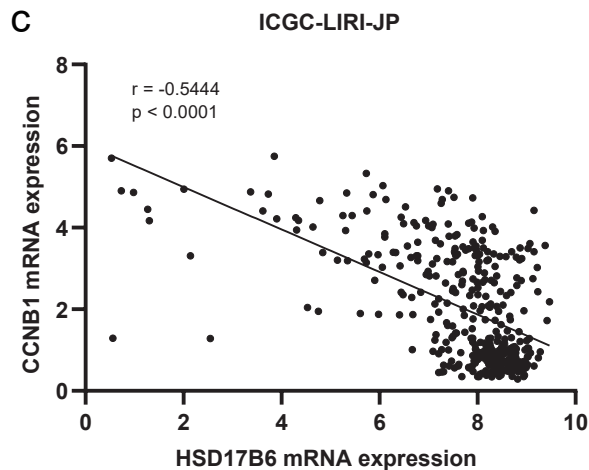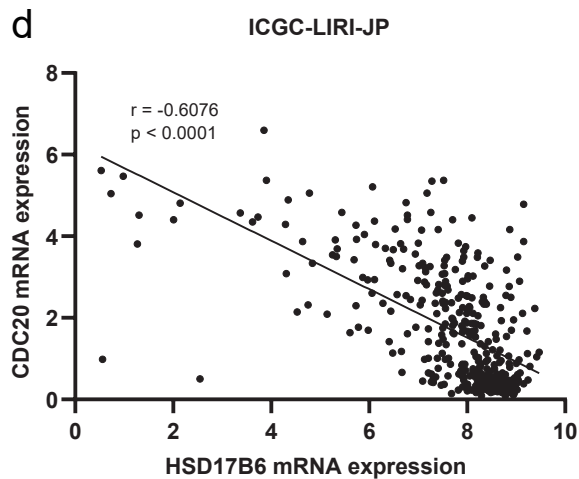

Supplement: Supplementary file 3 — Additional file 3: Fig. S2. Correlation of HSD17B6 expression with CCNB1/CDC20 expression in TCGA LIHC and ICGC-LIRI-JP datasets. [file 12935_2020_1298_MOESM3_ESM.pdf]
