# Supplementary material for: Downexpression of HSD17B6 correlates with clinical prognosis and tumor immune infiltrates in hepatocellular carcinoma
Source: Cancer Cell Int. 2020 Jun 3;20:210. doi: 10.1186/s12935-020-01298-5 (PMC7268300; doi:10.1186/s12935-020-01298-5)
Supplement: Supplementary file 4 — Additional file 4: Fig. S3. Correlation of HSD17B6 expression with infiltration level of immune cells by xCell in ICGC LIRI-JP dataset. [file 12935_2020_1298_MOESM4_ESM.pdf]

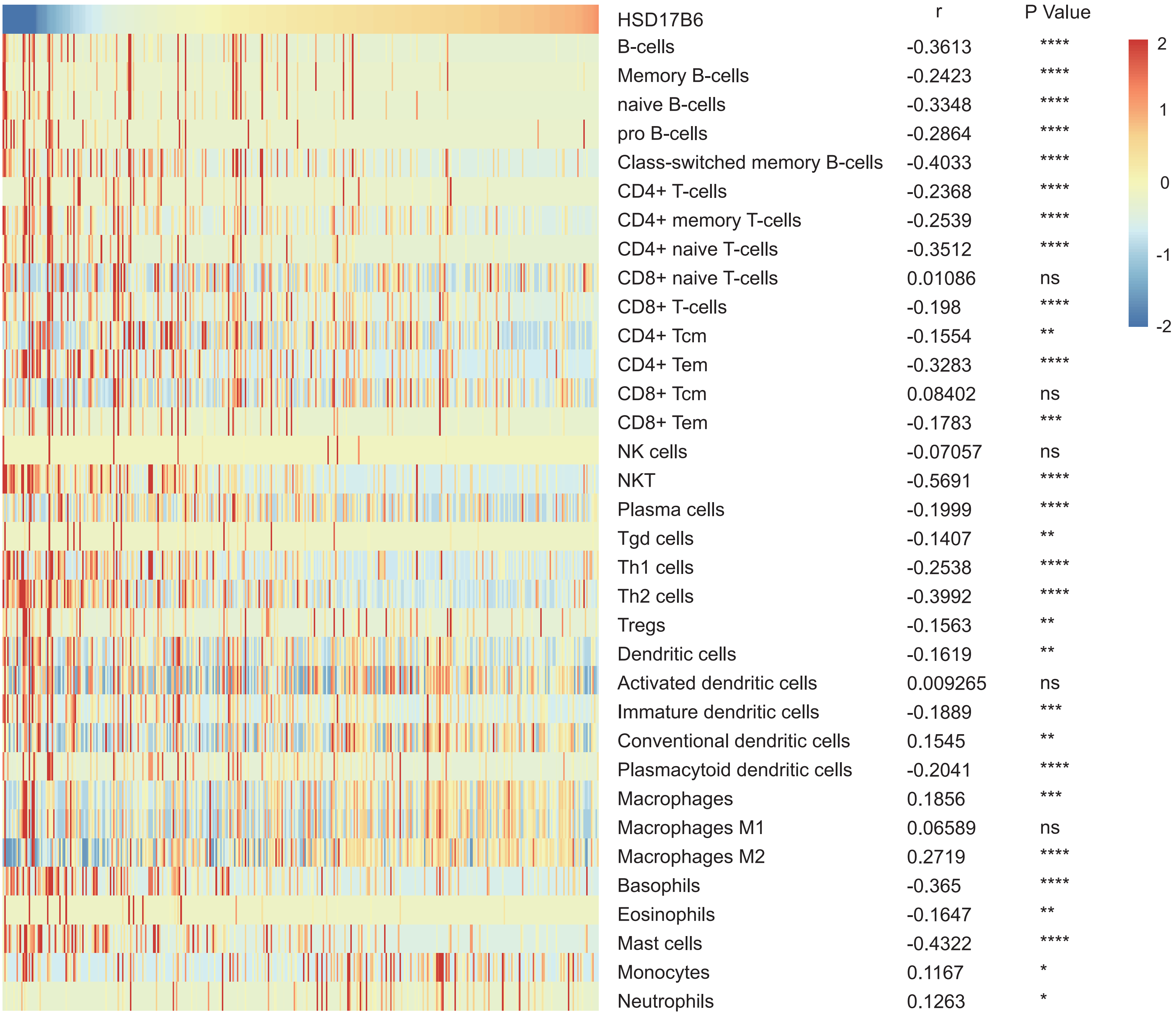
HSD17B6
r
P Value
B-cells -0.3613 ****
Memory B-cells -0.2423 ****
naive B-cells -0.3348 ****
pro B-cells -0.2864 ****
Class-switched memory B-cells -0.4033 ****
CD4+ T-cells -0.2368 ****
CD4+ memory T-cells -0.2539 ****
CD4+ naive T-cells -0.3512 ****
CD8+ naive T-cells 0.01086 ns
CD8+ T-cells -0.198 ****
CD4+ Tcm -0.1554 **
CD4+ Tem -0.3283 ****
CD8+ Tcm 0.08402 ns
CD8+ Tem -0.1783 ***
NK cells -0.07057 ns
NKT -0.5691 ****
Plasma cells -0.1999 ****
Tgd cells -0.1407 **
Th1 cells -0.2538 ****
Th2 cells -0.3992 ****
Tregs -0.1563 **
Dendritic cells -0.1619 **
Activated dendritic cells 0.009265 ns
Immature dendritic cells -0.1889 ***
Conventional dendritic cells 0.1545 **
Plasmacytoid dendritic cells -0.2041 ****
Macrophages 0.1856 ***
Macrophages M1 0.06589 ns
Macrophages M2 0.2719 ****
Basophils -0.365 ****
Eosinophils -0.1647 **
Mast cells -0.4322 ****
Monocytes 0.1167 *
Neutrophils 0.1263 *
2
1
0
-1
-2
